# Development and Psychometric Testing of an Infectious Disease Knowledge Questionnaire in a Convenience Sample

**DOI:** 10.3390/ijerph23030356

**Published:** 2026-03-11

**Authors:** Selda Seçginli, Nesrin İlhan, Gizemnur Torun, Merve Altıner Yaş, Seda Doğru Bolat

**Affiliations:** 1 Department of Nursing, Faculty of Health Sciences, Istanbul Atlas University, Istanbul 34408, Türkiye; 2 Department of Nursing, Faculty of Health Science, Istanbul Medeniyet University, Istanbul 34700, Türkiye; nesrin.ilhan@medeniyet.edu.tr; 3 Department of Nursing, Faculty of Health Science, Kocaeli University, Kocaeli 41380, Türkiye; gizemnur.torun@kocaeli.edu.tr; 4 Public Health Nursing Department, Faculty of Nursing, Istanbul University, Istanbul 34116, Türkiye; merve.altiner@istanbul.edu.tr; 5 Family Health Unit No. 09, Family Health Center No. 03, Hatay 31800, Türkiye; sedadogru12345@gmail.com

**Keywords:** community, infectious disease knowledge, psychometric testing, reliability, validity

## Abstract

**Highlights:**

**Public health relevance—How does this work relate to a public health issue?**

- This study addresses the need for a valid and reliable tool to assess the general population’s knowledge of infectious diseases, which is critical for designing effective public health interventions.
- By measuring community-level knowledge, the Infectious Diseases Knowledge Questionnaire (IDKQ) contributes to understanding public awareness gaps, which is essential for targeting prevention efforts and controlling disease transmission.

**Public health significance—Why is this work of significance to public health?**

- The development of the IDKQ is significant because it fills a gap in the availability of standardized tools for assessing infectious disease knowledge, particularly at the community level.
- With the rise of emerging infectious diseases, a tool that helps evaluate public knowledge can significantly inform health education initiatives and policies aimed at improving prevention and response strategies.

**Public health implications—What are the key implications or messages for practitioners, policymakers and/or researchers in public health?**

- For practitioners and policymakers, the IDKQ can serve as a practical instrument to evaluate the effectiveness of health education programs and identify areas where public knowledge needs improvement.
- Researchers can utilize the tool to assess community-level awareness across diverse populations, supporting global efforts to enhance public health preparedness and response to infectious disease outbreaks.

**Abstract:**

Objective: This study aimed to develop the Infectious Diseases Knowledge Questionnaire (IDKQ) and evaluate its psychometric properties for use in community settings. Methods: This methodological study was conducted with 533 adults aged ≥ 18 years. Data were collected using a sociodemographic information form and the IDKQ. Content validity was assessed by expert evaluation. Construct validity was examined using exploratory factor analysis (EFA) and confirmatory factor analysis (CFA). Reliability was evaluated through item–total correlations, internal consistency (KR-20), test–retest reliability, and intraclass correlation coefficients (ICC). Data analyses were performed using SPSS 25.0 and AMOS 21.0. Results: Content validity index values ranged from 0.94 to 1.00. EFA revealed a four-factor structure consisting of 17 items, explaining 45.66% of the total variance (KMO = 0.784; Bartlett’s test, *p* < 0.001). CFA demonstrated good model fit (χ^2^/df = 2.329, RMSEA = 0.074, CFI = 0.946, AGFI = 0.847, GFI = 0.887, SRMR = 0.045). The KR-20 coefficient was 0.735, the test–retest correlation was 0.604, and the ICC was 0.781. Conclusions: The IDKQ demonstrates acceptable internal consistency and moderate temporal stability, providing preliminary evidence of reliability and construct validity. It may serve as a tool for assessing infectious disease knowledge, although further validation in independent samples is recommended.

## 1. Introduction

Despite major advances in medicine and public health, infectious diseases remain a persistent threat to global public health [1,2,3,4]. Within an increasingly globalized world, their ability to transcend political and geographic boundaries places all populations at risk. The COVID-19 pandemic demonstrated the rapid global spread of infectious diseases and their profound impacts on health systems and socioeconomic structures, underscoring the need for holistic approaches to health promotion and disease prevention [2,5].

Globally, the spread of infectious diseases is influenced by social, economic, environmental, and structural factors, such as sanitation, safe water access, housing, education, mobility, and healthcare availability [6,7,8]. Climate change, extreme weather events, and disasters, combined with globalization, have intensified infectious disease transmission and increased the risk of outbreaks in communities [9,10,11,12]. In addition, natural disasters increase infectious disease risks by damaging infrastructure and creating conditions such as overcrowding, inadequate sanitation, and limited healthcare access [13,14].

The Sustainable Development Goals emphasize the prevention and control of infectious diseases as part of broader efforts to promote health and reduce inequalities worldwide [15,16]. Effective prevention strategies include clean water and sanitation, immunization, vector control, adequate nutrition, personal hygiene, and comprehensive disease management [17,18,19,20]. However, the success of these interventions is closely linked to individuals’ knowledge and understanding of infectious diseases [21]. The World Health Organization highlights continuous infection prevention and control education for both healthcare workers and communities as a cornerstone of global health promotion [22]. Health education and community-based awareness programs aimed at enhancing knowledge and attitudes towards infectious diseases will significantly prevent the spread of infectious diseases by increasing protective health behaviors in the community [21,23,24].

Evaluating individuals’ knowledge of infectious diseases is a crucial step in reducing transmission in the community. Such evaluations are essential for identifying health education needs and assessing the efficacy of existing educational programs [25]. A review of the literature revealed the absence of a standardized instrument specifically designed to assess the infectious disease knowledge of people in the community. Current instruments predominantly focus on measuring perceptions, attitudes, and awareness of infectious diseases [26,27]. This highlights a significant gap in assessing the knowledge of infectious diseases and evaluating the effectiveness of educational interventions. To accurately assess individuals’ knowledge levels and provide evidence-based data, it is necessary to develop a valid and reliable tool for evaluating the knowledge level of people regarding infectious diseases. Consequently, developing a valid and reliable instrument to measure community knowledge of infectious diseases is crucial. To our knowledge, there has been no Turkish language tool suitable for use in the community in Türkiye. This study aimed to develop an Infectious Disease Knowledge Questionnaire and conduct validity and reliability analyses.

## 2. Materials and Methods

### 2.1. Setting and Participants

This study was conducted as part of a project aimed at improving knowledge, awareness, and preventive behaviors related to infectious diseases in earthquake-affected areas. Data were collected between 20 January and 7 March 2025, from individuals living in container settlements in Hatay, Türkiye.

In scale development studies, a commonly recommended sample size is at least 10 participants per item [28]. According to Comrey and Lee, sample sizes of 300 are considered good and 500 are considered very good for psychometric analyses [29]. In this study, data were obtained from 533 participants, representing 59% of the total population living in the container settlements and meeting the recommended item-to-participant ratio for the 33-item questionnaire. The inclusion criteria were: age ≥ 18 years, literacy, absence of communication difficulties, and voluntary participation. Inclusion criteria for participants were: (1) aged 18 years or older, (2) living in container settlements and (3) having access to a smartphone and social media platforms (e.g., WhatsApp) to complete the questionnaire. Being a healthcare professional was defined as an exclusion criterion in the study.

To avoid double-dipping and ensure independent validation of the factor structure, Confirmatory Factor Analysis (CFA) was conducted on a separate dataset collected independently from the sample used for the Exploratory Factor Analysis (EFA). The CFA sample consisted of 243 adults residing in container settlements, recruited between 10 October and 22 December 2025. Data were collected using the same instruments and procedures applied during the EFA phase to maintain methodological consistency.

### 2.2. Data Collection

Data were collected using an online, self-administered questionnaire distributed via Google Forms^®^ through WhatsApp groups in container settlements. Participants were directed to the study link when they clicked the QR code. First, they read the consent form and selected the “I agree” option to confirm their participation in the study. Then, the questions became visible. The questionnaire required approximately 6–8 min to complete. The survey remained open for three weeks with three reminders sent. For test–retest reliability, a subset of participants (*n* = 50) was invited to complete the questionnaire again after two weeks. A total of 39 participants completed the second administration, resulting in a response rate of 78%.

### 2.3. Measures

Data were collected using a Sociodemographic Information Form and the Infectious Disease Knowledge Questionnaire. The sociodemographic form consisted of seven questions to determine the identifying characteristics of the participants, including age, sex, education status, marital status, employment status, income status, and perceived self-health status. This form was developed by researchers through a literature review [14,21,25,30].

The Infectious Disease Knowledge Questionnaire was developed to assess infectious disease knowledge among adults living in the community. A multi-phase scale development process was followed to ensure validity and reliability. A comprehensive literature review was conducted to identify domains relevant to infectious disease knowledge [6,18,30]. Consistent with DeVellis’s recommendation to generate a broad initial item pool [31], the conceptual framework was designed to comprehensively capture community-based infectious disease knowledge. The questionnaire was structured around eight domains: infectious diseases, general hygiene, personal hygiene, water hygiene, food hygiene, vector control, immunization, and symptoms of infectious diseases.

Based on the literature review, four independent researchers with expertise in public health nursing generated an initial pool of 76 items. Each researcher developed items independently, after which the items were reviewed collectively to eliminate duplication and redundancy. Through consensus meetings, the item pool was refined to a 35-item draft for expert evaluation. In line with methodological recommendations, the initial item pool was approximately three to four times larger than the intended final scale [31,32,33]. Items were formatted with three response options: “True”, “False”, and “I have no idea”. Nine items were reverse scored. Correct answers were scored as 1, while incorrect and “no idea” responses were scored as 0, yielding a total score range of 0–17, with higher scores indicating greater knowledge.

Content and face validity were evaluated by a panel of 18 experts, including public health nurses, public health nursing academicians, pediatric nurses, and infection control nurses. Experts assessed each item for relevance, clarity, and comprehensiveness using a 4-point scale (1 = not appropriate to 4 = completely appropriate). Items rated as 3 or 4 were considered acceptable. Based on expert feedback, revisions were made to item wording and structure, and redundant items were removed, resulting in a revised 33-item draft.

Readability refers to the ease or difficulty with which readers can understand a written text. Various mathematical readability formulas, primarily relying on syllable and word counts, have been developed to assess the complexity of written texts [34]. In this study, the Ateşman Readability Index was used, which was developed in 1997 based on the Flesch Reading Ease Formula and adapted to the grammatical structure of the Turkish language [35]. An online readability calculator (http://okunabilirlikindeksi.com/) was used to evaluate each item in the questionnaire. Scores derived from the Ateşman Readability Index were entered into an Excel spreadsheet and analyzed by the researchers.

The revised draft was pilot tested with 19 individuals representative of the target population. Participants reported that the items were clear and understandable, and no substantial modifications were required. The final version of the questionnaire consisted of 33 items.

### 2.4. Ethical Considerations

The study was approved by the Istanbul Atlas University Research Ethics Committee (Date: 21 August 2024, Approval no. 2024/07-05). Participants provided online consent after being informed of the study’s purpose, the confidentiality of their responses, and the voluntary nature of their participation.

### 2.5. Data Analysis

Data were analyzed using Statistical Package for the Social Sciences (SPSS) Version 25.0 and Analysis of Moment Structures (AMOS) Version 21.0 (SPSS Inc., Chicago, IL, USA). Sociodemographic data were examined by calculating frequencies, percentages, means, standard deviations, and minimum and maximum values. To evaluate item discrimination, an independent sample *t*-test was conducted comparing the responses of the 27% lowest- and highest-scoring groups. The instrument’s reliability was assessed using the Kuder-Richardson-20 (KR-20) coefficient. Item-to-total and inter-item correlations were determined using Pearson’s correlation coefficient. Test–retest reliability was analyzed using Spearman’s correlation, and intraclass correlation coefficients (ICC) were calculated. An item–total correlation of higher than 0.20 is considered desirable [36,37,38,39,40,41,42,43,44,45,46,47].

Item analyses were conducted within the Classical Test Theory (CTT) framework as a preliminary step prior to EFA. Because the instrument is a dichotomously scored knowledge test (true/false format), the initial analyses were conducted within the framework of Classical Test Theory (CTT). Item difficulty indices and corrected items–total correlations were calculated to evaluate item performance. Items with corrected items–total correlations below 0.20 were considered to demonstrate insufficient discrimination and were reviewed for removal. In knowledge tests, low discrimination suggests that an item does not adequately differentiate between individuals with higher and lower levels of overall knowledge. Consistent with recommendations in test development literature [31,48], poorly discriminating items were excluded prior to factor extraction.

Following this preliminary screening procedure, to examine validity, the content validity index, explanatory factor analysis, and confirmatory factor analysis were conducted on the remaining items to examine the latent factor structure. The Kaiser–Meyer–Olkin (KMO) test and Bartlett’s test of sphericity were used for factor analyses to ensure data adequacy. Exploratory factor analysis was conducted using Principal Component Analysis as the extraction method. The Kaiser criterion, scree-plot graph, and exploratory factor analysis were used to determine the number of factors. The rotated component matrix was formed using the Varimax rotation method, which maximizes the sum of the variances of the quadratic factor loads for each factor. The criteria for retaining an item was a minimum factor loading coefficient of 0.30. After the item analysis and EFA, the instrument was reduced to 17 items.

Confirmatory factor analysis was conducted to verify the created sub-dimensions. The CFA was performed using AMOS with the Maximum Likelihood estimation method. Model fit was evaluated using multiple goodness-of-fit indices, including the chi-square to degrees of freedom ratio (χ^2^/df), Root Mean Square Error of Approximation (RMSEA), Comparative Fit Index (CFI), Goodness of Fit Index (GFI), Adjusted Goodness of Fit Index (AGFI), and Root Mean Square Residual (RMSR).

## 3. Results

The mean age of the study participants was 33.59 ± 11.27 (min: 18; max: 71); 65.5% were women, 46.9% attended school for 18 years and below, 51% were married, 56.7% were working and 63.4% had a moderate-income level. More than half (52.7%) of the participants reported being in good health (Table 1).

### 3.1. Reliability

The reliability assessment of Kuder-Richardson 20 revealed a robust correlation of 0.735 (within the range of 0.55–0.63) (Table 2). The arithmetic mean values of the items ranged from 0.42 ± 0.49 to 0.97 ± 0.18, respectively. It was determined that the items were sufficient to provide item discrimination power with a 27% lower-upper group analysis (*p* < 0.01). The corrected item–total correlation coefficients were positive and varied between 0.037 and 0.40, respectively. Items with correlation values less than 0.20 were removed, starting from the lowest value. Thus, 10 items that did not correlate completely with the scale were removed from the draft scale. The stability of the instrument was examined using test–retest reliability with 39 participants at 2-week intervals. The test–retest correlation coefficient for the instrument was 0.60, and the intraclass correlation coefficient (ICC) was 0.78. The Spearman correlation coefficients of the sub-dimensions ranged from 0.49, 0.48, 0.64, and 0.69, respectively. For the sub-dimensions, the ICC values were 0.63, 0.72, 0.78, and 0.90, respectively (Table 3).

### 3.2. Validity

#### 3.2.1. Content Validity

The CVI was calculated to determine the validity of the IDKQ. The experts evaluated the items of the IDKQ, with a ratio ranging from 0.94 to 1.00. The Scale-Level CVI of the IDKQ items was determined as 0.99.

#### 3.2.2. Construct Validity

Exploratory factor analysis demonstrated sampling adequacy (KMO = 0.784) and a significant Bartlett’s Test of Sphericity (χ^2^ = 1698.854, df = 253, *p* < 0.001). Communalities ranged from 0.178 to 0.606, and six items with factor loadings below 0.30 were removed. The analysis identified a four-factor structure comprising 17 items with eigenvalues greater than 1.

**Figure 1:**
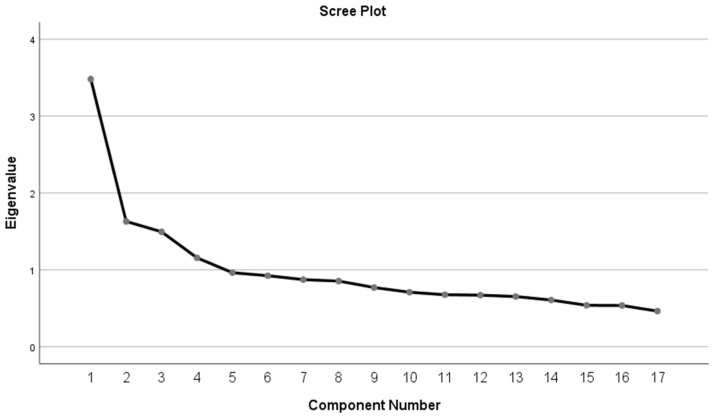
Scree plot from the exploratory factor analysis of the 17-item Infectious Diseases Knowledge Questionnaire.

Based on eigenvalues, total variance explained, and the scree plot, a four-factor structure was confirmed. In the item analysis, 10 items were removed, leaving 23 items to be included in the exploratory factor analysis. Next, EFA was performed on the remaining items. During this stage, items with communality (extraction) values below 0.30 were considered to have insufficient contribution to shared variance and were removed. Accordingly, 4 items (6, 12, 13, and 15) were first excluded. After repeating the factor analysis, two additional items (18 and 23) were removed because they showed low communality values and weak contributions to the factor structure. Thus, the EFA process resulted in the removal of 6 items, and the final instrument consisted of 17 items (Supplementary Files S1 and S2). The scree plot presented in Figure 1 depicts the retained 17 items after EFA for clarity; the initial extraction included all 33 items, and the plot correctly reflects the point at which factors were retained based on eigenvalues and the scree criterion. Factor loadings ranged from 0.391 to 0.778, and the four factors explained 45.66% of the total variance (Table 4).

The factors were labeled as Infectious Diseases and Misinformation, Personal and Environmental Hygiene, Immunization and Personal Protection, and Water- and Vector-borne Diseases, and the scree plot is presented in Figure 1.

**Figure 2:**
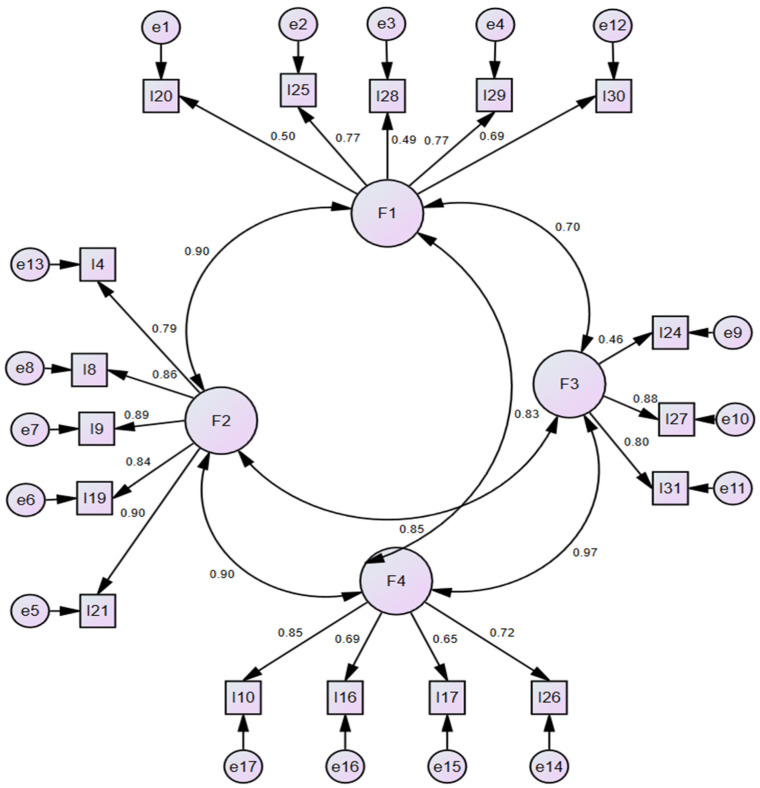
Structural model from the confirmatory factor analysis of the 17-item Infectious Diseases Knowledge Questionnaire (*n* = 243).

A measurement model that included the four factors obtained as a result of the EFA and the items that comprised these factors was formed. The model fit indices were χ^2^/df = 2.329, RMSEA = 0.074, AGFI = 0.847, GFI = 0.887, SRMR = 0.045, and CFI = 0.946 (Table 5). The factor loadings were between 0.49 and 0.77 in the first sub-dimension, between 0.79 and 0.90 in the second sub-dimension, between 0.46 and 0.88 in the third sub-dimension, and between 0.65 and 0.85 in the fourth sub-dimension (Figure 2).

Table 5 shows the fit criteria of the model as a result of the confirmatory factor analysis. In our study, the Root Mean Square Error of Approximation (RMSEA), Goodness of Fit Index (GFI), and Adjusted Goodness of Fit Index (AGFI) were acceptable for the model of the scale in the CFA result; the χ^2^/df, Standardized Root Mean Square Residual (SRMR), and Comparative Fit Index (CFI) are a good fit.

In this study, the readability assessment using the Atesman Readability Formula showed that the average reading level for all items was 64.1 ± 16.04. This score shows that the questionnaire items are generally moderately easy to read [35].

## 4. Discussion

Knowledge of infectious diseases and their transmission pathways is key to controlling their dissemination in the community. A reliable and valid tool for assessing infectious disease knowledge can help identify information needs and evaluate the effectiveness of educational programs, thereby supporting the development of effective strategies. However, no tool is available to assess this concept in the community. This is the first study to develop a measurement tool to assess knowledge of infectious diseases among people living in the community. In this study, the Infectious Diseases Knowledge Questionnaire was developed, and its psychometric properties were tested. The results of this study provide preliminary evidence supporting the reliability and validity of the IDKQ, consisting of 17 items.

The most fundamental step in scale development is to define the conceptual and theoretical characteristics of the trait to be measured [38]. In this study, a comprehensive literature review was performed to identify the key topics and the appropriate format for inclusion in the questionnaire. An initial pool of items was then developed, content validation was evaluated by experts, readability was evaluated, and a pilot test was conducted.

Measurement tools must possess validity and reliability characteristics to provide accurate and standardized measurements [31,32,33]. The reliability of the IDKQ was evaluated using both item analysis and test–retest procedures. Preliminary item screening ensured that only items with adequate discriminative power were retained, consistent with best practices in knowledge test development [36,37]. Test–retest analysis showed moderate stability over time, which aligns with findings from similar community-based knowledge assessments [31,32]. While the internal consistency was acceptable, the moderate test–retest reliability indicates that the instrument should be applied with some caution when used for repeated measurements. Overall, these results suggest that the IDKQ is a psychometrically sound tool for assessing infectious disease knowledge, with further validation warranted in broader or different populations.

In this study, the internal consistency of the questionnaire was assessed using the KR-20 coefficient. The KR-20 method is appropriate for test items scored dichotomously [39]. The results indicated that the questionnaire is sufficiently reliable for evaluating infectious disease knowledge. While some sub-dimensions showed relatively lower consistency, this is consistent with previous research suggesting that subscales with few items and dichotomous response formats often yield lower reliability estimates [28,40]. Overall, the findings support the use of the IDKQ as a psychometrically sound instrument, although caution may be warranted when interpreting scores for specific sub-dimensions.

Upon examining the test–retest mean scores of the IDKQ, no statistically significant differences were observed between the pre- and post-test scores across any sub-dimensions, indicating the scale’s temporal stability. The literature suggests that a correlation of at least moderate (r ≥ 0.40) and ideally high (r ≥ 0.60) is expected for stability over time [41]. According to the Spearman correlation analysis, the IDKQ sub-dimensions demonstrated moderate temporal stability. The correlation coefficient for the total score of the scale was 0.604, which reflects moderate and statistically significant stability over time. These findings suggest that the IDKQ yields reliable and consistent results. Furthermore, the Intraclass Correlation Coefficient (ICC), another measure of reliability over time, was calculated. The ICC is a statistic that comprehensively evaluates consistency between measurements. According to the classification by Koo and Li (2016), ICC values between 0.50 and 0.74 are considered moderate, between 0.75 and 0.89 are deemed good, and ≥0.90 are regarded as excellent reliability [42]. The IDKQ sub-dimensions demonstrated moderate-to-excellent reliability over time, and the total score showed good overall reliability, indicating that the questionnaire provides consistent and stable measurements. The test–retest reliability was assessed using both Pearson correlation (r = 0.604) and the intra-class correlation coefficient (ICC = 0.781). The discrepancy arises because the ICC considers both agreement and systematic shifts in scores, whereas Pearson correlation assesses only rank-order consistency. In this case, participants’ relative ordering remained moderately consistent, but small shifts in absolute scores reduced the Pearson r. Therefore, while the ICC indicates acceptable reliability in terms of agreement, caution is warranted in interpreting individual score stability. Overall, these findings suggest that the IDKQ demonstrates moderate-to-good temporal stability. Given that the instrument was designed as a community-based knowledge assessment tool rather than a clinical diagnostic instrument, this level of stability may be considered acceptable.

Validity refers to the degree to which a measurement tool can accurately measure the characteristic that is intended to be measured [43]. In this study, validity was examined in two ways. First, the content validity was assessed by a panel of experts. Second, construct validity was assessed using exploratory and confirmatory factor analyses. Content validity assesses whether the items in the scale represent the area to be measured [31,36,37]. For this purpose, the content validity of each item was reviewed by experts. Expert evaluations using Davis’s technique and the Content Validity Index indicated excellent content validity for the IDKQ, with high agreement among experts confirming that the items appropriately represent the construct being measured [44].

Exploratory Factor Analysis and Confirmatory Factor Analysis were employed to assess the construct validity of the questionnaire. The Kaiser-Meyer-Olkin test and Bartlett’s test of sphericity were conducted to evaluate the adequacy of the sample size and the suitability of the data for factor analysis [45,46]. In the study, the KMO measure indicated that the sample was adequate for conducting factor analysis, supporting the appropriateness of the data for exploring the questionnaire’s underlying structure [46]. Following confirmation of the sample and data suitability for factor analysis, EFA was conducted using Principal Component Analysis (PCA) with varimax rotation. In line with standard psychometric practices, items with low factor loadings were removed to strengthen the questionnaire’s construct validity, ensuring that all retained items meaningfully contributed to the underlying factors [47,48]. The literature suggests that the number of factors should be determined when the eigenvalue is ≥1 [49]. The Scree plot test was used to determine the number of factors in the study. The EFA revealed a four-factor structure comprising 17 items with an eigenvalue greater than 1, explaining 45.66% of the total variance. The EFA supported a multi-factor structure for the IDKQ, consistent with theoretical expectations and previous studies. The observed structure indicates that the items cluster meaningfully into distinct factors, providing preliminary evidence of the questionnaire’s construct validity within this population. Although the four-factor structure explained 45.66% of the total variance, it indicates that the current 17-item instrument may not capture all dimensions of infectious disease knowledge. In knowledge-based instruments, some potentially relevant domains might not be fully represented. Therefore, while the current findings provide preliminary support for construct validity, future studies may expand the content scope to improve explanatory power.

Subsequently, a CFA was conducted to evaluate the validity of the scale’s factor structure. The confirmatory factor analysis indicated that the proposed model fits the data well, with all fit indices falling within acceptable ranges [50]. These results provide further support for the construct validity of the IDKQ. In this context, the results obtained demonstrate that the four-factor structure of the questionnaire is statistically significant and valid. All fit indices fell within the recommended range, indicating that the model exhibited a good fit and that the scale possessed good construct validity. These results suggest the strong validity of the items in the Infectious Diseases Knowledge Questionnaire.

It is essential for individuals in the community to thoroughly comprehend the content of the IDKQ. Therefore, readability was assessed using the Ateşman Readability Index. In the study, the average readability score of all items was 64.1 ± 16.04. This finding indicates that the questionnaire items are suitable for use at the community level, suggesting that the instrument can be understood by individuals with varying educational backgrounds and levels of health literacy.

This study has several limitations. First, the reduction in the number of items during the validation process may have restricted the content coverage. In addition, the use of convenience sampling limits the generalizability of the findings to the broader population. Because data were collected through an online survey, selection bias may have occurred. The sample was predominantly female and included a disproportionately high number of university graduates and individuals with moderate income compared to the general population of container settlements. This distribution likely reflects disparities in digital access, as individuals with internet access and higher educational attainment were more likely to participate. These participants may differ from the general population in terms of psychological stress, living conditions, and recent exposure to public health messages. Therefore, the findings may not be generalizable to the broader community.

Test–retest reliability was evaluated using a relatively small subsample (*n* = 39), yielding a correlation coefficient of 0.604. Although this level of stability may be acceptable for a knowledge assessment instrument, it may be insufficient for clinical applications. The moderate Pearson correlation suggests that some individual scores may have shifted between test administrations, highlighting the preliminary nature of this instrument’s temporal stability. Further studies with larger test–retest samples are recommended to confirm these findings.

Although the IDKQ was designed to cover multiple domains of infectious disease knowledge, its general scope may limit its applicability to disease-specific contexts. The tool is intended primarily as a population-level screening and monitoring tool to identify general knowledge gaps, rather than as a diagnostic or clinically prescriptive measure. Finally, items with low item–total correlations were removed prior to exploratory factor analysis as part of a preliminary screening process. While this approach is consistent with classical test theory–based scale development practices, it may have reduced the likelihood of identifying subtle or emerging factors characterized by low-discrimination items.

## 5. Conclusions

The findings indicate that the Infectious Diseases Knowledge Questionnaire demonstrates acceptable internal consistency and moderate test–retest reliability, providing preliminary evidence of construct validity. Notably, the IDKQ is the first tool specifically developed to assess the general population’s knowledge of infectious diseases. The questionnaire items are suitable for use at the community level. It may be a concise, feasible, and practical measurement instrument for researchers and health care providers. Given the significant public health burden of emerging and re-emerging infectious diseases worldwide, the availability of valid and reliable measurement tools is essential for informing evidence-based public health interventions and policy decisions. It can be used for evaluating knowledge levels before and after interventions, particularly in the context of planning health education programs and initiatives aimed at improving individuals’ knowledge. Moreover, the tool provides a standardized measure that can be adapted for use in diverse community settings and epidemiological studies, supporting international research efforts aimed at improving public health preparedness and response. Further validation in independent samples is recommended.

## Figures and Tables

**Table 1:** Sociodemographic characteristics of the study participants, January–March 2025 (*n* = 533).

Characteristics	Mean ± SD	
Age (Years)	33.59 ± 11.27 (18–71)	
	*n*	%
Gender		
Female	349	65.5
Male	184	34.5
Educational status		
Literate but no formal schooling	16	3.0
Elementary school graduate	45	8.4
Middle school graduate	35	6.6
High school graduate	154	28.9
University	254	47.7
Master’s/PhD graduate	29	5.4
Marital status		
Married	272	51.0
Single	230	43.2
Widowed/divorced	31	5.8
Employment status		
Employed	302	56.7
Not employed	231	43.3
Income status		
Very poor	17	3.2
Poor	78	14.6
Moderate	338	63.4
Good	92	17.3
Very good	8	1.5
Health assessment		
Very poor	7	1.3
Poor	20	3.8
Average	199	37.3
Good	281	52.7
Very good	26	4.9

Source: Authors’ own work.

**Table 2:** Total and subdimension scores of the Infectious Diseases Knowledge Questionnaire, January-March 2025 (*n* = 533).

Sub-Dimensions	Mean ± SD	Median	Min–Max	KR-20
Infectious Diseases and Misinformation	2.63 ± 1.54	3	0	0.620
Personal and Environmental Hygiene	4.5 ± 0.84	5	0	0.548
Immunization and Personal Protection	2.84 ± 0.5	3	0	0.625
Water and Vector-borne Diseases	3.25 ± 0.96	4	0	0.545
IDKQ Total	13.23 ± 2.74	14	0–17	0.735

IDKQ: Infectious Diseases Knowledge Questionnaire; KR-20: Kuder-Richardson.

**Table 3:** Test–retest reliability analysis of the Infectious Diseases Knowledge Questionnaire, January-March 2025 (*n* = 39).

Sub-Dimensions	Pre-Test Mean ± SD	Post-Test Mean ± SD	Z	*p*	r	*p*	ICC
Infectious Diseases and Misinformation	2.28 ± 1.41	2.62 ± 1.44	−1.519	0.129	0.492	0.001	0.625
Personal and Environmental Hygiene	4.59 ± 0.55	4.74 ± 0.50	−1.897	0.058	0.479	0.002	0.722
Immunization and Personal Protection	2.92 ± 0.27	2.92 ± 0.27	0.000	1.000	0.639	0	0.780
Water and Vector-borne Diseases	3.49 ± 0.88	3.46 ± 0.91	−0.302	0.763	0.687	0	0.902
IDKQ Total	13.28 ± 2.20	13.74 ± 2.09	−1.819	0.069	0.604	0	0.781

IDKQ: Infectious Diseases Knowledge Questionnaire; Z: Wilcoxon Signed Ranks Test; r: Spearman’s correlation coefficient; ICC: Interclass correlation coefficient. Source: Authors’ own work.

**Table 4:** Exploratory factor analysis of the 17-item Infectious Diseases Knowledge Questionnaire, January-March 2025 (*n* = 533).

Items	Factor Loadings			
	Factor 1	Factor 2	Factor 3	Factor 4
Item 25—A single dose of rabies vaccine is sufficient after animal bites such as those from cats or dogs.	0.614			
Item 20—Crimean-Congo hemorrhagic fever (CCHF) is transmitted by cat bites and can be fatal.	0.613			
Item 30—The clothes of a person with scabies should be washed at 40 °C.	0.607			
Item 28—Antibiotics must always be used to treat infectious diseases.	0.581			
Item 29—In scabies, it is sufficient to treat only the infected person.	0.518			
Item 4—Sharing items such as towels, pillows, and bedding can transmit infectious diseases.		0.391		
Item 8—There is no need to wash hands if there is no visible dirt.		0.698		
Item 21—Garbage bags can be left open until the garbage is disposed of.		0.636		
Item 9—Tap water is safe to drink after a natural disaster.		0.633		
Item 19—Cats and dogs do not transmit infectious diseases unless they bite.		0.557		
Item 31—To maintain a strong immune system, daily sleep duration should be 6–8 h.			0.778	
Item 27—The nose and mouth should be covered with a tissue or the inside of the elbow when coughing or sneezing.			0.751	
Item 24—Childhood vaccinations are necessary to protect against diseases such as measles, chickenpox, and hepatitis.			0.635	
Item 10—Unclean drinking or tap water can cause diseases such as diarrhea, typhoid, and cholera.				0.720
Item 17—Malaria and West Nile virus are transmitted by mosquitoes.				0.670
Item 26—Fluid intake should be increased in cases of diarrhea and vomiting.				0.455
Item 16—Consumption of raw milk and unpasteurized dairy products can lead to brucellosis.				0.446
Eigenvalue	3.481	1.63	1.495	1.157
Explained variance (%)	12.735	11.915	11.033	9.978
Total explained variance (%)	45.661			
KMO	0.784			
Bartlett’s χ^2^ (*p*)	1292.770 (*p* < 0.001)			

KMO: Kaiser Meyer Olkin. Source: Authors’ own work.

**Table 5:** Confirmatory factor analysis of the 17-item Infectious Diseases Knowledge Questionnaire (*n* = 243).

**Model**	**χ^2^/df**	**RMSEA**	**CFI**	**AGFI**	**GFI**	**RMSR**
	2.329	0.074	0.946	0.847	0.887	0.045

χ^2^ (chi square): difference between model and data; df: degrees of freedom; GFI: goodness-of-fit index; AGFI: adjusted goodness-of-fit index; CFI: comparative fit index; RMSEA: root mean square error of approximation; RMSR: root mean square residual. Source: Authors’ own work.

## Data Availability

The data presented in this study are available upon request from the corresponding author.

## Supplementary Materials

The following supporting information can be downloaded at: https://www.mdpi.com/article/10.3390/ijerph23030356/s1, File S1: Items of the Turkish version of Initial 33-Item Pool of Infectious Disease Knowledge Questionnaire (IDKQ); File S2: Turkish version of the Final 17-Item Infectious Disease Knowledge Questionnaire (IDKQ).

## Abbreviations

The following abbreviations are used in this manuscript:

IDKQ	Infectious Diseases Knowledge Questionnaire
EFA	Exploratory Factor Analysis
CFA	Confirmatory Factor Analysis
KR-20	Kuder-Richardson 20
ICC	Intraclass Correlation Coefficients
SPSS	Statistical Package for the Social Sciences
AMOS	Analysis of Moment Structures
RMSR	Root Mean Square Residual
RMSEA	Root Mean Square Error of Approximation
CFI	Comparative Fit Index
GFI	Goodness of Fit Index
AGFI	Adjusted Goodness of Fit Index
PCA	Principal Component Analysis
KMO	Kaiser Meyer Olkin
CVI	Content Validity Index
