# Supplementary material for: Development and Psychometric Testing of an Infectious Disease Knowledge Questionnaire in a Convenience Sample
Source: Int J Environ Res Public Health. 2026 Mar 11;23(3):356. doi: 10.3390/ijerph23030356 (PMC13027333; doi:10.3390/ijerph23030356)
Supplement: Supplementary file 1 [file ijerph-23-00356-s001.zip › ijerph-4154746-supplementary.pdf]

Supplementary File 1: Items of the Turkish version of Initial 33-Item Pool of Infectious Disease Knowledge Questionnaire (IDKQ)

Items and Turkish Version	Status	Decision Rationale
1. The risk of infectious diseases increases during natural disasters such as earthquakes and floods.(True)<br>*Deprem, sel gibi doğal afet durumlarında bulaşıcı hastalık riski artar.*	Removed	Corrected item–total correlation < .20
2. Diseases such as tuberculosis, whooping cough, and influenza spread easily in crowded environments.(True)<br>*Tüberküloz (verem), boğmaca, grip gibi hastalıklar kalabalık ortamlarda kolay bulaşır.*	Removed	Corrected item–total correlation < .20
3. Contact of open wounds with concrete, wood, metal, and soil surfaces causes tetanus. (True)<br>*Açık yaraların beton, ahşap, metal ve toprak yüzeylerle teması tetanoza neden olur.*	Removed	Corrected item–total correlation < .20
4. Sharing items such as towels, pillows, and bedding can transmit infectious diseases. (True)<br>*Havlu, yastık ve yatak gibi eşyaların ortak kullanımı bulaşıcı hastalıklara neden olur.*	Retain	-
5. Toilet, bathroom, and kitchen surfaces should be cleaned by mixing half a teacup (50 mL) of bleach into 10 liters of water.(False)<br>*Tuvalet, banyo ve mutfak temizliği, 10 litre suya yarım çay bardağı (50 ml) çamaşır suyu karıştırılarak yapılmalıdır.*	Removed	Corrected item–total correlation < .20
6. To prevent infectious diseases, hands should be washed with soap and water for at least 10 seconds.(False)<br>*Bulaşıcı hastalıkları önlemek için eller su ve sabunla en az 10 saniye yıkanmalıdır.*	Removed	Communality < .30
7. If soap and water are not available, hands can be cleaned using an alcohol-based hand disinfectant. (True)<br>*Su ve sabun olmadığında eller alkol içeren dezenfektanla ovuşturularak temizlenebilir.*	Removed	Corrected item–total correlation < .20
8. There is no need to wash hands if there is no visible dirt. (False)<br>*Ellerde gözle görünür kirlilik yok ise yıkamaya gerek yoktur.*	Retained	-
9.Tap water is safe to drink after a natural disaster. (False)<br>*Doğal afet sonrası musluk suyu içilebilir.*	Retained	-
10. Unclean drinking or tap water can cause diseases such as diarrhea, typhoid, and cholera. (True)<br>*Temiz olmayan içme ve kullanma suları ishal, tifo, ve kolera gibi hastalıklara yol açabilir.*	Retained	-
11.Water of uncertain cleanliness should be boiled for at least 10 minutes before drinking. (False)<br>*Temizliğinden emin olunmayan suların içilebilmesi için en az 10 dakika kaynatılması gerekir.*	Removed	Corrected item–total correlation < .20
12. Cooked food can be safely consumed after being left at room temperature for 5–6 hours. (False).<br>*Pişmiş yiyecekler oda sıcaklığında 5-6 saat bekletilerek tüketilebilir.*	Removed	Communality < .30
13. When preparing food, vegetables should be chopped before meat products on cutting boards.(False)	Removed	Communality < .30

Yiyecek hazırlamak için kullanılan kesme tahtalarında önce et ürünleri sonra sebzeler doğranabilir.		
14. The ideal refrigerator storage temperature for food should be 6–8°C. (False)<br>*Gıdaların buzdolabında saklanması için ideal sıcaklık 6-8 derece olmalıdır.*	Removed	Corrected item–total correlation < .20
15. Frozen foods should be thawed by leaving them at room temperature. (False)<br>*Dondurulmuş gıdalar oda sıcaklığında bekletilerek çözdürülmelidir.*	Removed	Communality < .30
16. Consumption of raw milk and unpasteurized dairy products can lead to brucellosis. (True)<br>*Çiğ süt ve ürünleri brusella hastalığına yol açabilir.*	Retained	-
17. Malaria and West Nile virus are transmitted by mosquitoes. (True)<br>*Sıtma ve Batı Nil Virüsü sivrisinekler ile bulaşan hastalıklardır.*	Retained	-
18. To combat flies, water should not be left in open containers such as flowerpots, water containers, and pet food bowls. (True)<br>*Sineklerle mücadele için saksı, bidon ve kedi, köpek mama kapları gibi ağzı açık kaplarda su bulundurulmamalıdır.*	Removed	Low communality and weak contributions to the factor structure
19. Cats and dogs do not transmit infectious diseases unless they bite.(False)<br>*Kedi ve köpekler ısırmadığı sürece bulaşıcı hastalığa neden olmaz.*	Retained	-
20. Crimean-Congo hemorrhagic fever (CCHF) is transmitted by cat bites and can be fatal. (False)<br>Kırım Kongo Kanamalı Ateşi (KKKA), kedi ısırığı ile bulaşan ve *ölümle sonuçlanabilen bir hastalıktır.*	Retained	-
21. Garbage bags can be left open until the garbage is disposed of. (False)<br>*Evdeki çöpler atılana kadar çöp poşetlerinin ağzı açık kalabilir.*	Retained	-
22. If you have contact with someone with measles, you should receive a measles vaccine within the first 3 days. (True).<br>*Kızamık hastası ile temas durumunda ilk 3 gün içerisinde kızamık aşısı olmak gerekir.*	Removed	Corrected item–total correlation < .20
23. You should receive a tetanus vaccine after sustaining an injury.(True)<br>*Yaralanmalardan sonra tetanoz aşısı yaptırmak gerekir.*	Removed	Low communality and weak contributions to the factor structure
24. Childhood vaccinations are necessary to protect against diseases such as measles, chickenpox, and hepatitis.(True)<br>*Kızamık, suçiçeği ve hepatit gibi hastalıklardan korumak için çocukluk çağı aşılarını yaptırmak gerekir.*	Retained	-
25. A single dose of rabies vaccine is sufficient after animal bites such as those from cats or dogs. (False)<br>*Kedi, köpek gibi hayvan ısırıklarından sonra 1 doz kuduz aşısı olmak yeterlidir.*	Retained	-
26. Fluid intake should be increased in cases of diarrhea and vomiting.(True).<br>*İshal ve kusma durumunda su tüketimi arttırılmalıdır.*	Retained	-
27. The nose and mouth should be covered with a tissue or the inside of the elbow when coughing or sneezing.(True)<br>*Öksürürken veya hapşırırken burun ve ağız bir mendille veya dirseğin içi ile kapatılmalıdır.*	Retained	-
28. Antibiotics must always be used to treat infectious diseases. (False)	Retained	-

Bulaşıcı hastalıkların tedavisi için mutlaka antibiyotik kullanmak gerekir.		
29. In scabies, it is sufficient to treat only the infected person.(False). *Uyuz hastalığında, hasta kişinin tedavi olması yeterlidir.*	Retained	-
30. The clothes of a person with scabies should be washed at 40°C. (False) *Uyuz olan kişinin çamaşırları 40 derecede sıcaklıkta yıkanmalıdır.*	Retained	-
31. To maintain a strong immune system, daily sleep duration should be 6-8 hours.(True) *Bağışıklık sistemini güçlü tutmak için günlük uyku süresi 6-8 saat olmalıdır.*	Retained	-
32. To keep the immune system strong, you should exercise at least 100 minutes per week. (False) *Bağışıklık sistemini güçlü tutmak için haftada en az 100 dakika egzersiz yapılmalıdır.*	Removed	Corrected item–total correlation < .20
33. To keep the immune system strong, you should drink 8–10 glasses of water every day. (True) *Bağışıklık sistemini güçlü tutmak için her gün 8-10 bardak su içilmelidir.*	Removed	Corrected item–total correlation < .20

Supplementary File 2: Turkish version of the Final 17- Item Infectious Disease Knowledge Questionnaire (IDKQ)

Maddeler		Doğru	Yanlış	Bilmiyorum
1	Kedi, köpek gibi hayvan ısırıklarından sonra 1 doz kuduz aşısı olmak yeterlidir. (Yanlış)			
2	Kırım Kongo Kanamalı Ateşi (KKKA), kedi ısırığı ile bulaşan ve ölümle sonuçlanabilen bir hastalıktır. (Yanlış)			
3	Uyuz olan kişinin çamaşırları 40 derecede sıcaklıkta yıkanmalıdır. (Yanlış)			
4	Bulaşıcı hastalıkların tedavisi için mutlaka antibiyotik kullanmak gerekir. (Yanlış)			
5	Uyuz hastalığında, hasta kişinin tedavi olması yeterlidir. (Yanlış)			
6	Havlu, yastık ve yatak gibi eşyaların ortak kullanımı bulaşıcı hastalıklara neden olur. (Doğru)			
7	Ellerde gözle görünür kirlilik yok ise yıkamaya gerek yoktur. (Yanlış)			
8	Evdeki çöpler atılana kadar çöp poşetlerinin ağzı açık kalabilir. (Yanlış)			
9	Doğal afet sonrası musluk suyu içilebilir. (Yanlış)			
10	Kedi ve köpekler ısırmadığı sürece bulaşıcı hastalığa neden olmaz. (Yanlış)			
11	Bağışıklık sistemini güçlü tutmak için günlük uyku süresi 6-8 saat olmalıdır. (Doğru)			
12	Öksürürken veya hapşırırken burun ve ağız bir mendille veya dirseğin içi ile kapatılmalıdır. (Doğru)			
13	Kızamık, suçiçeği ve hepatit gibi hastalıklardan korumak için çocukluk çağı aşılarını yaptırmak gerekir. (Doğru)			
14	Temiz olmayan içme ve kullanma suları ishal, tifo, ve kolera gibi hastalıklara yol açabilir. (Doğru)			
15	Sıtma ve Batı Nil Virüsü sivrisinekler ile bulaşan hastalıklardır. (Doğru)			
16	İshal ve kusma durumunda su tüketimi arttırılmalıdır. (Doğru)			
17	Çiğ süt ve ürünleri brusella hastalığına yol açabilir. (Doğru)			
